# Expanding Opportunities for Professional Development: Utilization of Twitter by Early Career Women in Academic Medicine and Science

**DOI:** 10.2196/11140

**Published:** 2018-07-23

**Authors:** Jaime D Lewis, Kathleen E Fane, Angela M Ingraham, Ayesha Khan, Anne M Mills, Susan C Pitt, Danielle Ramo, Roseann I Wu, Susan M Pollart

**Affiliations:** ^1^ Department of Surgery College of Medicine University of Cincinnati Cincinnati, OH United States; ^2^ Department of Emergency Medicine Temple University Hospital Philadelphia, PA United States; ^3^ Department of Surgery School of Medicine and Public Health University of Wisconsin Madison, WI United States; ^4^ Department of Emergency Medicine Stanford University School of Medicine Stanford, CA United States; ^5^ Department of Pathology School of Medicine University of Virginia Charlottesville, VA United States; ^6^ Weill Institute for Neurosciences Department of Psychiatry University of California San Francisco San Francisco, CA United States; ^7^ Department of Pathology and Laboratory Medicine Perelman School of Medicine University of Pennsylvania Philadelphia, PA United States; ^8^ Department of Family Medicine School of Medicine University of Virginia Charlottesville, VA United States

**Keywords:** female, leadership, social media, academic success, professional development

## Abstract

The number of women entering medical school and careers in science is increasing; however, women remain the minority of those in senior faculty and leadership positions. Barriers contributing to the shortage of women in academics and academic leadership are numerous, including a shortage of role models and mentors. Thus, achieving equity in a timelier manner will require more than encouraging women to pursue these fields of study or waiting long enough for those in the pipelines to be promoted. Social media provides new ways to connect and augments traditional forms of communication. These alternative avenues may allow women in academic medicine to obtain the support they are otherwise lacking. In this perspective, we reflect on the role of Twitter as a supplemental method for navigating the networks of academic medicine. The discussion includes the use of Twitter to obtain (1) access to role models, (2) peer-to-peer interactions, and continuous education, and (3) connections with those entering the pipeline—students, trainees, and mentees. This perspective also offers suggestions for developing a Twitter network. By participating in the “Twittersphere,” women in academic medicine may enhance personal and academic relationships that will assist in closing the gender divide.

## Introduction

The number of women pursuing seats in medical schools continues to increase, and women comprise nearly one-half of applicants and more than half of matriculants [1,2]. These statistics remain stable throughout medical school and residency; however, just over one-third of junior faculty in academic medicine are women [1]. Women in senior faculty and leadership are even less common, with women holding only 22.8% of full professor, 15% of permanent chair, and 16% of dean positions [1,3]. In science, technology, engineering, and mathematics (STEM) disciplines, women are less likely to be hired into any faculty position than men and less likely to be retained in appointments in mathematics [4]. Though the number of women entering medical and STEM careers has been steady for some years, advancement to leadership positions continues to lag. The dearth of women in these top positions is no longer a “pipeline” issue [5].

## Challenges Experienced by Women in Academic Medicine

Women who leave academics early in their careers cite multiple concerns, including inequalities in compensation, lack of role models, difficulties achieving work-life integration, effects of unconscious and implicit bias on experiences, frustrations in research including funding gaps, and a non-collaborative, and biased work environment [6,7]. In academic medicine, women continue to receive lower salaries and women researchers receive smaller startup packages and less funding from organizations such as the National Institutes of Health, have fewer opportunities to publish, and are promoted at slower and lower rates [8-10]. Though initiatives are being developed at multiple levels to help advance and promote women to leadership positions, these concerns persist for women attempting to climb the ladders in academic medicine [11]. Women must consider supplemental approaches to obtain necessary skills for career advancement and to navigate the networks and societies that are central to academic success.

The Association of American Medical Colleges (AAMC), whose mission it is to “improve the nation's health through the advancement of academic medicine” [12], is attempting to bridge the gender gap in academic medicine by providing yearly leadership retreats for women faculty at the early and mid-career levels. Retreats are designed to help women learn to “navigate the academic medicine enterprise as well as continue on the path to leadership” [13]. At the 2017 AAMC Early Career Women Faculty Leadership Development Seminar, the authors of this article connected through Twitter engagement by using the meeting hashtag, #EWIMS (Early Career Women in Medicine and Science). This multidisciplinary group of junior faculty, along with one senior conference faculty member, then met in person to discuss our struggles and successes as women in academic medicine. Our conversation about the impact of Twitter on our professional lives led to the conclusion that social media can fill some of the gaps in support that women in academic medicine endure, and it gave rise to this collaborative piece. In our experience, the Twittersphere is a unique resource for fostering relationships within and outside of medicine as it expands critical planes of engagement through (1) access to role models potentially leading to mentorship and sponsorship, (2) networking, peer-to-peer support, and education, and (3) connection with students, trainees, and mentees.

## Access to Mentorship and Sponsorship

Mentorship is important for increasing diversity at the top and identifying a role model who “looks like you” makes it easier to follow suit [14,15]. Several excellent avenues exist to provide mentorship for early career women faculty. Institutions and departments frequently offer formal mentoring programs. Conferences such as the AAMC Women’s Leadership Seminars (@AAMCMeetings) or groups like the American Medical Women’s Association (@AMWAdoctors) strive to connect women in academic medicine with support, mentorship, potential sponsors, and opportunity [16,17]. However, the capacity of leadership, senior faculty, and these programs to connect with junior faculty are limited by factors such as distance, time, finances, low mentor to mentee ratio, and availability.

Educators have also realized the need to draw on informal learning networks to complement formal supports and institutions and have turned to Twitter and other social media platforms as extensions of the learning environment [18]. Relationships developed on Twitter may help fill the gaps in more traditional methods of face-to-face role modeling, mentoring, and sponsorship [19,20]. One #EWIMS participant described presenting her poster to a prominent physician at a conference. Their relatively formal interaction quickly became warm and jovial the instant the two recognized one another from prior interactions on Twitter. That afternoon, they met for coffee to discuss the #EWIMS participant’s career plans and are now collaborating on a review paper. Others have described collegial relations formed on Twitter as “some of the strongest professional relationships” they developed [21].

The value of access to a large pool of same-gender role models, mentors, and sponsors accessible on Twitter may be particularly important in situations where these relationships are not uniformly available to junior faculty [22-25]. For example, women in cardiac and thoracic surgery hold well under 10% of faculty positions in leading academic facilities [26]. While it is unlikely that women in these fields will hold positions at the same institution, they can identify one another and build relationships online (@WomenInThoracic). Similar relationships and their value in professional development have been described by public health professionals who have connected despite geographic distance and learners experiencing the “3C’s of Twitter”—Community, Communication, and Casual (informal) Learning—and contribute positively to building social capital [20,27,28].

In addition, as men currently hold the majority of leadership positions, it is imperative that women identify and build relationships with men who are gender inclusive in supporting the development of leaders [14,29]. Professional organizations may unknowingly maintain an isolating and gender-divisive atmosphere during after-hours events that can prove a barrier to women attempting to develop relationships with leaders in their fields. Twitter provides an alternate forum for faculty to connect with leadership outside of gendered-organized spaces. Movements such as #HeforShe highlight this need as well as identify those who are willing participants [29]. Though the 280-character messages provided on Twitter are unlikely to constitute substantive mentoring, the platform sets the stage for rising faculty to access more senior role models with whom they might not otherwise have the opportunity to connect. These connections may then promote informal and formal mentorship and sponsorship assisting in the advancement of women physicians [30].

## Peer Support and Education

Intra- and interdisciplinary peer-to-peer connections may develop through participation in Twitter-based movements, chats, journal clubs, and other educational platforms. These allow individuals to create personal learning networks (PLNs), systems of “interpersonal connections and resources that support informal learning” [20,21,31]. Teachers have used similar PLNs as accessible and transformative alternatives to traditional modules for professional development [21].

Physicians across the country and worldwide have met virtually and in person and garnered support from participation in #ILookLikeASurgeon, #NYerORCoverChallenge, #WomenInMedicine, and #MeTooMedicine [32,33]. Several chat groups focused on challenges and opportunities experienced by women in medicine meet regularly. These include #AWSchat hosted by @WomenSurgeons and #WomenInMedicine hosted by @womeninmedchat. Twitter users can follow their professional organizations, such as the American College of Surgeons (@AmCollSurgeons) for up to date information on advocacy and other news, and journals such as Journal of the American Medical Association (@JAMA_current), and the New England Journal of Medicine (@NEJM) for the latest breaking research. Each of these illustrates Twitter as an environment for networked learning and development of a community of practice, common in the field of education offline, and online [34,35].

There are many hashtags devoted to teaching vignettes (often image based as those that involve pathology and radiology, #radpath) and journal clubs like general surgery (#UMichSurgJC), trauma surgery (#EASTjc), Association of Women Surgeons (#AWSchat), nephrology (#nephJC), and pathology (#pathJC). Sharing conference material with the use of a specific hashtag allows interested parties to follow meeting content and participate in the conversation regardless of physical presence at the event. For example, the #EWIMS hashtag facilitated connectivity for the varied communities of conference attendees, faculty, and worldwide followers. In addition to ease of participation, an international group of researchers recently showed that the perceived costs of conference participation via social media were far lower than live attendance while nearly half of respondents felt that they would learn the same or more from the social media-based format [36]. When following conferences, “backchannels” may form leading to the identification of and deeper conversation with others of similar interests [37].

## Connections to Students, Trainees, and Mentees

Twitter also provides a platform from which to reach and inspire girls and women who are students and trainees, especially those who might not otherwise have access to women role models. Social media can help faculty to connect with talented students, residents, and others for inspiration, promotion of program and institution, and recruitment. Pearls of wisdom and other messages are shared broadly among communities of medical educators and learners using hashtags such as #DearFutureMD, #MedEd, and #SurgEd. Students in other fields, including art and history, have also realized the value of access to multiple educators and peers outside of their classrooms to provide feedback and leading them to explore national organizations, institutions, and publications that they otherwise may not have been aware [35]. Among medical students participating in social media use curriculum, positive experience including a mechanism for connecting and sharing was expressed and a trend towards personal growth including a more humanistic approach to patients [38].

One example of how Twitter can facilitate such a relationship includes an #EWIMS participant who was contacted by a high school student who lived over a thousand miles away. This connection led to a Skype Chat with the student’s all-female medicine interest club, a behind-the-scenes tour of the student’s local academic medical center arranged by the #EWIMS participant, and ongoing mentorship via Twitter, and other social media outlets.

## Limitations to the Use of Twitter

Like any tool, there are challenges when utilizing Twitter for the above pursuits. Messages must be concise, despite a recent doubling of the length, as tweets are limited to 280 characters. Most of this work is often performed before or after hours and on personal devices as many organizations block the use of social media sites [20,21]. Those who use Twitter often rate themselves higher on measures of general technology proficiency [21].

While popular among many, the reach of Twitter is limited as only 23% of active American internet users (or 20% of the adult population) have a Twitter account [39]. Also, the use of Twitter declines by over 50% in users over the age of 50, a limitation in access to more experienced role models, mentors, and sponsors [40]. As the “net generation” continues to age, we expect to see significant changes in these statistics [38,41].

Some consider Twitter to be “frivolous, superficial, or dangerous” and do not find professional value in social media [21,42]. Other barriers to use include concerns regarding cyberbullying, time requirements, privacy concerns, and the blurring of lines between learners and educators as well as health professionals and patients, inappropriate sharing of protected health information and violation of confidentiality, and potential risks of sharing misinformation or something that is (perceived as) unethical or unprofessional [21,38,43]. Among nonusers of social media in health professional education, many cited a lack of understanding of how to integrate use, lack of departmental support and technical skills, and uncertainty of departmental policies [41]. Social media-based research often lacks in methodological rigor [44]. However, there are numerous resources available regarding the responsible use of social media, and some medical schools are incorporating principles of use into the curriculum and promotion criteria for faculty [38,45-48].

## A Call to Action

With unprecedented numbers of women entering medical and science careers, there has never been a more important or realistic opportunity to enhance the field’s emphasis on retaining women in academic medicine. Twitter’s ability to connect women faculty in academic medicine with others surviving and thriving in the field can facilitate and promote long-term success in a challenging career environment.

We suggest the following strategies for developing a supportive environment through the use of Twitter. First, promote a culture of Tweeting among academics*.* Medical schools should support the use of Twitter among faculty, such as including professional social media activity as one component of the faculty dossier. Profile pages of faculty at schools of medicine should publish Twitter handles, allow faculty to integrate Twitter feed on their profile pages, and encourage faculty Twitter engagement. Schools may also report journal article influence using metrics, such as the Altmetric, that measure the social media impact of research articles. When faculty publish articles, they should be encouraged to share through Twitter and discouraged from believing that it is shameless self-promotion. Second, facilitate connections. Faculty on Twitter should make a point of acknowledging participation as part of routine networking, education, and mentoring activities (eg, conferences, lectures). In academic presentations, faculty should include Twitter handles (not just email addresses) as contact information. As part of mentoring and leadership development activities, mentors and sponsors should be encouraged to use social media and to support their trainees in doing so responsibly. Training programs should include workshops on how to get the most out of Twitter, facilitated by faculty seasoned in its use.

Closing the sustained gender gap in hiring, promoting, and retaining women in academic medicine will certainly require a multi-faceted approach. As the authors of this piece have experienced, the use of Twitter as part of this approach can facilitate ties fundamental to the long-term success of women in medicine and science. We are encouraged by the growing flurry of tweets that have become the roar of many academic women in medicine and science advancing through their careers.

## Abbreviations

AAMC: Association of American Medical Colleges
EWIMS: Early Career Women in Medicine and Science
PLN: personal learning network
STEM: science, technology, engineering, and mathematics
